# Fabrication of Amine-Modified Magnetite-Electrochemically Reduced Graphene Oxide Nanocomposite Modified Glassy Carbon Electrode for Sensitive Dopamine Determination

**DOI:** 10.3390/nano8040194

**Published:** 2018-03-27

**Authors:** Quanguo He, Jun Liu, Xiaopeng Liu, Guangli Li, Dongchu Chen, Peihong Deng, Jing Liang

**Affiliations:** 1 School of Materials Science and Energy Engineering, Foshan University, Foshan, 528000, China; hequanguo@126.com (Q.H.); liu.jun.1015@163.com (J.L.); 2 College of Life Science and Chemistry, Hunan University of Technology, Zhuzhou, 412007, China; amituo321@163.com (X.L.); liangjingabbey@126.com (J.L.); 3 Department of Chemistry and Material Science, Hengyang Normal University, Hengyang, 421008, China; dph1975@163.com (P.D.)

**Keywords:** NH_2_–Fe_3_O_4_ nanoparticles, reduced graphene oxide, modified electrode, dopamine, electrochemical oxidation

## Abstract

Amine-modified magnetite (NH_2_–Fe_3_O_4_)/reduced graphene oxide nanocomposite modified glassy carbon electrodes (NH_2_–Fe_3_O_4_/RGO/GCEs) were developed for the sensitive detection of dopamine (DA). The NH_2_-Fe_3_O_4_/RGO/GCEs were fabricated using a drop-casting method followed by an electrochemical reduction process. The surface morphologies, microstructure and chemical compositions of the NH_2_–Fe_3_O_4_ nanoparticles (NPs), reduced graphene oxide (RGO) sheets and NH_2_–Fe_3_O_4_/RGO nanocomposites were characterized by scanning electron microscopy (SEM), transmission electron microscopy (TEM), X-Ray diffraction (XRD) and Fourier-transform infrared (FTIR) spectroscopy. The electrochemical behaviors of DA on the bare and modified GCEs were investigated in phosphate buffer solution (PBS) by cyclic voltammetry (CV). Compared with bare electrode and RGO/GCE, the oxidation peak current (*i_pa_*) on the NH_2_–Fe_3_O_4_/RGO/GCE increase significantly, owing to the synergistic effect between NH_2_–Fe_3_O_4_ NPs and RGO sheets. The oxidation peak currents (*i_pa_*) increase linearly with the concentrations of DA in the range of 1 × 10^−8^ mol/L – 1 × 10^−7^ mol/L, 1 × 10^−7^ mol/L – 1 × 10^−6^ mol/L and 1 × 10^−6^ mol/L – 1 × 10^−5^ mol/L. The detection limit is (4.0 ± 0.36) ×10^−9^ mol/L (*S*/*N* = 3). Moreover, the response peak currents of DA were hardly interfered with the coexistence of ascorbic acid (AA) and uric acid (UA). The proposed NH_2_–Fe_3_O_4_/RGO/GCE is successfully applied to the detection of dopamine hydrochloride injections with satisfactory results. Together with low cost, facile operation, good selectivity and high sensitivity, the NH_2_–Fe_3_O_4_/RGO/GCEs have tremendous prospects for the detection of DA in various real samples.

## 1. Introduction

Dopamine (DA) is one of the most important neurotransmitters, playing a key role in the regulation of the functions of the renal, hormonal, the central nervous and cardiovascular systems [1]. Abnormal levels of DA may cause serious neurological disorders such as Huntington’s disease, Parkinson’s disease and schizophrenia [1,2,3]. Generally, the basal DA concentration in the central nervous system is 10^−6^ mol/L – 10^−8^ mol/L. Therefore, it is essential to develop a low cost, good selectivity and high sensitivity method for the detection of DA at the physiological level. Traditional techniques including high performance liquid chromatography [4,5], mass spectrometry [6], fluorescent spectrometry [7,8] and electrochemoluminescence [9,10] have been widely used for the detection of DA. Although these methods possess high precision and reliability, they often involve complicated and time-consuming experimental protocols with expensive instrumentations. Due to its low cost, facile operation, high selectivity and sensitivity, the electrochemical method has received growing attention in biosensors recently [11,12]. As is well known, bare glassy carbon electrodes (GCE) have always suffered from serious problems such as interference and electrode fouling, which can result in poor selectivity and reproducibility. To resolve these problems, various chemicals (including conducting polymers, metal or metal oxide nanomaterials and carbon-based nanomaterials) and modified electrodes have been developed for the sensitive detection of DA [13,14,15,16,17,18].

Magnetite nanoparticles (Fe_3_O_4_ NPs) are widely used in the biomedical field due to their good biocompatibility, large surface area, excellent magnetic target properties and distinct catalytic activity [19,20,21,22,23]. The advantages of Fe_3_O_4_ NPs are their low cost, ease of preparation and excellent water solubility [24]. More importantly, they have excellent electronic and magnetic properties due to electron transfer between Fe^3+^ and Fe^2+^ [25]. However, Fe_3_O_4_ NPs have poor electrical conductivity and inclination of agglomeration, which will eventually cause poor sensing performances with a narrow linear range and limited detection limits [26]. The aforementioned problem can be effectively solved by compositing the Fe_3_O_4_ NPs with graphene. Owing to its excellent electrical conductivity, large surface-to-volume ratio and rapid heterogeneous electron transfer rate, graphene has been considered one of the most promising materials for electrochemical sensors in recent years [27]. As a result, a Fe_3_O_4_/graphene composite combines the individual merits of each component, including large surface area, excellent electrical conductivity and distinct catalytic activity, which can be used to detect dopamine, hydrogen peroxide, guanosine and N-acetylcysteine [24,28,29,30,31].

Amine-modified Fe_3_O_4_ (NH_2_–Fe_3_O_4_) can bind with graphene-type through hydrogen bonds between the amino-groups attached to NH_2_–Fe_3_O_4_ and the available carboxylic groups of graphene-type. Consequently, the NH_2_–Fe_3_O_4_/graphene-type nanocomposite has good dispersibility and stability. Recently, the NH_2_–Fe_3_O_4_/graphene-type has been widely used for the removal of environmental pollutants such as heavy metal ions due to their strong adsorb ability and ease of separation from waste water [32,33,34]. In our previous report, the oxidation of DA is an adsorption-controlled electrochemical process [35]. Therefore, NH_2_–Fe_3_O_4_/graphene-type nanocomposites are expected to enhance the adsorption of DA on the electrode and improve the sensing performance. However, to our best knowledge, only a few papers have reported NH_2_–Fe_3_O_4_/graphene-type toward the sensitive detection of DA. Wu and coworkers prepared an NH_2_–Fe_3_O_4_ NPs/graphene-type modified glassy carbon electrode (Fe_3_O_4_/Gr/GCE) toward DA detection, combining the advantages of NH_2_–Fe_3_O_4_ NPs and chemically reduced graphene oxide. The proposed electrode displayed a linear range of 0.2–38 μM and a detection limit of 0.126 μM [24]. For the purpose of detecting DA at the physiological level (10^−6^ mol/L – 10^−8^ mol/L), the dynamic range and detection limits still need to be further improved. Moreover, chemical reduction is not eco-friendly since it requires some poisonous reductants such as hydrazine and metal hydride [36]. In this case, the oxygen-containing groups have almost been removed from graphene due to the use of strong reductant, which is not favorable for the electrochemical oxidation of dopamine [37,38].

Inspired by Wu’s work, we developed NH_2_–Fe_3_O_4_/RGO nanocomposites modified GCE (NH_2_–Fe_3_O_4_/RGO/GCE) for detecting DA at the physiological level. In contrast to Wu’s work, the NH_2_–Fe_3_O_4_/RGO/GCE is prepared by way of drop-casting the NH_2_–Fe_3_O_4_/RGO dispersion on the bare GCE and subsequently undergoing a facile electrochemical reduction of GO process. In contrast, the electrochemical reduction method is green and eco-friendly due to the elimination of the reductants. Moreover, the electrochemical performance can be easily regulated by electrochemical reduction conditions (including reduction potential as well as time). Second-order derivative linear sweep voltammetry (SDLSV) shows higher sensitivity and selectivity as compared to the other electrochemical methods such as differential pulse voltammetry (DPV) and square wave voltammetry (SWV) [39]. Hence, SDLSV was employed to detect the DA samples. The morphologies, microstructure and chemical composition of NH_2_–Fe_3_O_4_ NPs, RGO and NH_2_–Fe_3_O_4_/RGO were characterized by scanning electron microscopy (SEM), transmission electron microscopy (TEM), X-Ray diffraction (XRD) and Fourier-transform infrared (FTIR) spectroscopy accordingly. Then the cyclic voltammetry (CV) behaviors of DA on the surface of bare and modified GCEs were also investigated. Moreover, the detection conditions including pH, scan rate, accumulation potential as well as time were further optimized. The sensing performances in terms of anti-interference, linear range and detection limit of DA on the NH_2_–Fe_3_O_4_/RGO/GCEs were evaluated systematically. Finally, the proposed NH_2_–Fe_3_O_4_/RGO/GCEs were used to detect DA in real samples.

## 2. Experimental Section

### 2.1. Materials and Chemicals

Graphite powder, sodium nitrate (NaNO_3_), concentrated sulfuric acid (H_2_SO_4_), potassium permanganate (KMnO_4_), hydrogen peroxide (H_2_O_2_), ferric trichloride hexahydrate (FeCl_3_·6H_2_O), sodium acetate anhydrous (NaAc), 1,2-ethylenediamine (ED), ethylene glycol (EG), potassium ferricyanide (K_3_Fe(CN)_6_), potassium ferrocyanide (K_4_Fe(CN)_6_), potassium nitrate (KNO_3_), phosphoric acid (H_3_PO_4_), sodium hydroxide (NaOH), hydrochloric acid (HCl) and ethyl alcohol were purchased from Sinopharm Chemical Reagent Co., Ltd. (Shanghai, China). DA was purchased from Sigma-Aldrich Co. (St. Louis, CA, USA). All these reagents were used as received without further purification. A stock solution of 1.0 × 10^−3^ mol/L DA was prepared by dissolving DA in deionized water (DI water) and stored at 4 °C when not in use. Standard solutions for calibration curves were prepared by appropriate dilution of the stock solution with DI water.

### 2.2. Synthesis of NH_2_–Fe_3_O_4_ NPs

Amine-modified Fe_3_O_4_ NPs were synthesized by solvothermal method according to previous report [24]. In brief, 1.0 g of FeCl_3_·6H_2_O was dissolved in 20 mL of EG. Then, 3 g of NaAc, 0.4 g NaOH and 10 mL of ED were added into the solution and vigorously stirred under ultrasound for 30 min. Afterwards the mixture was transferred to 30 mL Teflon-lined stainless-steel autoclave and autoclaved at 200 °C for 8 h. After cooling down to room temperature naturally, the black precipitates (NH_2_–Fe_3_O_4_ NPs) were obtained with an external magnetic field and washed with DI water and ethyl alcohol alternately. Finally, the resulting products were dispersed in DI water to obtain 1 mg/mL NH_2_–Fe_3_O_4_ NPs solution.

### 2.3. Synthesis of NH_2_–Fe_3_O_4_/GO Nanocomposites 

Graphene oxide (GO) was prepared using the modified Hummers’ method [40]. Typically, 0.5 g of graphite powder and 0.5 g of NaNO_3_ were slowly added into 23 mL cooled concentrated H_2_SO_4_ under vigorous mechanically stirring. Then, 3.0 g of KMnO_4_ were gradually added into the mixture with continuous stirring under ice bath. Afterwards, the mixture was transferred to 35 °C water bath and stirred for 2 h. The reaction was terminated by adding 100 mL of DI water into the mixture. The mixture added into 20 mL of 30% H_2_O_2_ aqueous solution in batches, which turned the color form dark golden yellow. The as-obtained suspension was filtered washed with 150 mL of hydrochloric acid (1:10) and 150 mL of DI water repeatedly and then vacuum-dried at 50 °C overnight to obtain graphite oxide. 100 mg of graphite oxide was dispersed in 100 mL DI water and exfoliated to GO by ultrasonication for 2 h. It was then centrifuged at 6000 rpm for 30 min to remove unexfoliated graphite oxide and excess graphite. Finally, 1 mL of NH_2_–Fe_3_O_4_ NPs solution (1 mg/mL) was added into 20 mL of GO solution (1 mg/mL) under ultrasound exposure for 2 h and the NH_2_–Fe_3_O_4_/GO nanocomposite dispersion were obtained.

### 2.4. Fabrication of NH_2_–Fe_3_O_4_/RGO/ GCE

Prior to electrode modification, the GCE was polished to form mirror-like surface using α-Al_2_O_3_ with different fine sizes (1.0 μm, 0.3 μm and 0.05 μm), then continuously ultrasonicated in ethyl alcohol and DI water (each for 1 min). The NH_2_–Fe_3_O_4_/GO/GCE were fabricated via drop-casting of 5 μL NH_2_–Fe_3_O_4_/GO dispersion on the surface of GCE and dried under infrared lamp radiation. The NH_2_–Fe_3_O_4_/GO/GCEs were finally obtained by electrochemical reduction of GO. Then NH_2_–Fe_3_O_4_/RGO/GCE was prepared by electrochemical reducing of GO in NH_2_–Fe_3_O_4_/GO/GCE at a suitable constant potential for a period in PBS (pH 6.5). The optimum reduction potential as well as time was also explored.

### 2.5. Characterization

The surface morphologies and microstructure of NH_2_–Fe_3_O_4_ NPs, RGO and NH_2_–Fe_3_O_4_/RGO were characterized by scanning electron microscopy (SEM), transmission electron microscopy (TEM) and Powder X-ray diffraction (XRD). The SEM images were obtained from a Hitachi S-3000N scanning electron microscope (Hitachi, Tokyo, Japan) at an acceleration potential of 30 kV. The TEM images were taken on a JEOL JEM-2010 (HT, Tokyo, Japan) operated at 200 kV. XRD patterns were operated with an X-ray diffractometer (PANalytical, Holland) operating at 40 kV and 40 mA with Cu Kα radiation (λ = 0.1542 nm). Samples were scanned in the 2θ range of 10°–70° with scan rate of 0.05°s^−1^. The amine-doping sample was characterized by Fourier transform infrared spectroscopy (FTIR, Varian Excalibur 3100 spectrometer, Palo Alto, CA, USA) with the wavenumber range of 500–4000 cm^−1^ and a resolution of 1 cm^−1^. The electrochemical experiments were carried out on a CHI660E electrochemical workstation (Chenhua Instrument Co. LTD, Shanghai, China) and a Polarographic Analyzer (JP-303E, Chengdu Instrument factory, Chengdu, China). Unless otherwise stated, 0.1 mol/L phosphate buffer saline (PBS) was used as the supporting electrolyte.

### 2.6. Electrochemical Experiments

Cyclic voltammetry (CV) and second-order derivative linear sweep voltammetry (SDLSV) were performed with a standard three-electrode system. Bare or modified GCEs was used as working electrode, platinum wire electrode and saturated calomel electrode (SCE) was acted as auxiliary electrode and reference electrode, respectively. The electrochemical active areas of bare and modified GCEs were estimated using CV recorded in a freshly prepared [Fe(CN)_6_]^3−/4−^ solution (5.0 × 10^−4^ mol/L). The CV behavior of DA on NH_2_–Fe_3_O_4_/RGO/GCE was measured in a 10 mL electrochemical cell containing 5.0 × 10^−5^ mol/L DA and 0.1 mol/L PBS. SDLSV was used to detect DA due to its high resolution and sensitivity. Both the CVs and SDLSVs were recorded at a scan rate of 100 mV/s, after a suitable accumulation period under stirring at 500 rpm and a 5 s rest. The potential scan ranges of both the CV and SDLSV were 0–1.0 V. Before electrochemical detection, pure N_2_ was bubbled through the standard solution of DA to remove O_2_ dissolved the solution.

### 2.7. Analysis of Real Samples

Dopamine hydrochloride injections were purchase from Aladdin Reagent Co. (Shanghai, China). 2 mL dopamine hydrochloride injections (containing 2 mg dopamine hydrochloride) were diluted to 100 mL with 0.1 M PBS (pH 3.5) to obtain DA diluents. Then DA diluent was further diluted with 0.1 M PBS (pH 3.5) to prepare DA samples of various concentration. Under the optimal detection conditions, the content of dopamine in the dopamine samples was detected using SDLSV by standard addition method.

## 3. Result and Discussion

### 3.1. Optimization of Electrochemical Reduction Conditions

**Figure 1:**
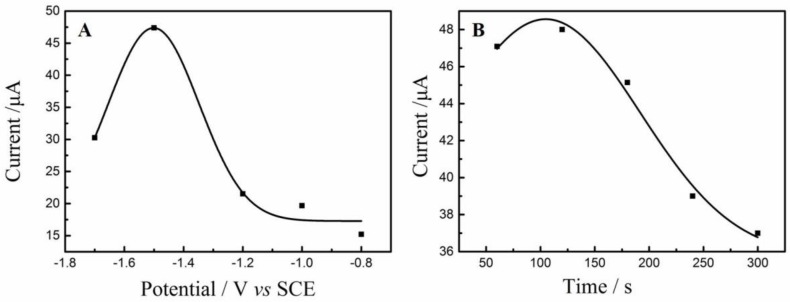
Optimization of reduction potential (**A**) and reduction time; (**B**) on the oxidation peak currents (*i_pa_*) for graphene oxide (GO) reduction.

The reduction potential as well as time is two crucial parameters for GO reduction. Firstly, the NH_2_–Fe_3_O_4_/RGO/GCEs were fabricated under various reduction potential for 300 s and then the oxidation peak currents (*i_pa_*) of as-obtained electrodes were compared. As shown in Figure 1A, the maximal *i_pa_* is obtained when the reduction potential is at −1.5 V. The reduction degree of GO increases with the reduction potential shifts from −0.8 V to 1.5 V. Accordingly, the electrical conductivity of NH_2_–Fe_3_O_4_/RGO/GCE may increase due to the restoration of conductive carbon-conjugate structure [41]. However, the *i_pa_* decrease when the reduction potential shifts to more negative direction, probably because the oxygen-containing groups closely relating to dispersibility [42] and electrocatalytic active sites are almost removed. Furthermore, the reduction time was optimized with the electrochemical reduction of −1.5 V. The largest *i_pa_* is obtained when the reduction time is 120 s (Figure 1B). There is no doubt that the reduction degree of GO increased over time. However, the *i_pa_* decreased with the reduction time beyond 120 s, since the oxygen-containing groups were almost removed due to excessive reduction of GO. Thus, the optimal reduction conditions are suggested as −1.5 V and 120 s.

### 3.2. Characterization of NH_2_–Fe_3_O_4_/RGO Nanocomposites

**Figure 2:**
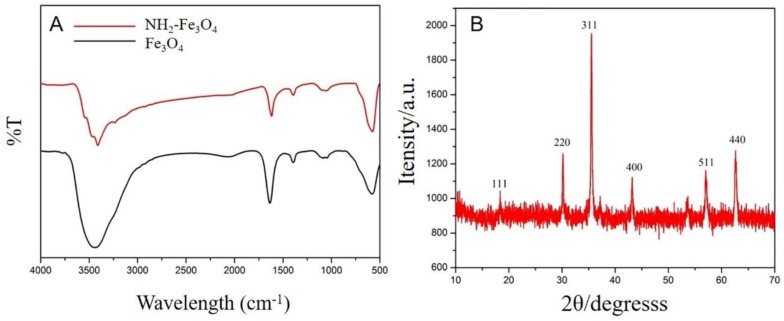
(**A**) Fourier-Transform Infrared (FTIR) spectra of Fe_3_O_4_ and NH_2_–Fe_3_O_4_; (**B**) the X-Ray Diffraction (XRD) pattern of Fe_3_O_4_ NPs.

Figure 2A shows the FTIR spectra of Fe_3_O_4_ NPs and NH_2_–Fe_3_O_4_ NPs. In the FTIR spectra of Fe_3_O_4_ NPs, the broad band in the range 3300–3600 cm^−1^ is due to the stretching vibrations of –OH, which is also appointed to the OH^−^ absorbed by Fe_3_O_4_ nanoparticles. In the FTIR spectra of NH_2_–Fe_3_O_4_ NPs, strong absorption peak at 577 cm^−1^ is also due to the stretching vibrations of Fe–O bond of Fe_3_O_4_ NPs. The absorption bands near 3211 cm^−1^ and 1637 cm^−1^ appear due to the vibration of –OH and there also exists the contribution of –NH for the band near 3211 cm^−1^. Moreover, the peak intensity of stretching vibrations of Fe–O bond is weakened, suggesting Fe_3_O_4_ NPs modified with –NH_2_ successfully. The Fe_3_O_4_ NPs were further characterized by XRD (Figure 2B). The diffraction peaks located at 2θ of 19.7°, 30.4°, 35.7°, 43.2°, 57.3° and 62.8° are corresponding to (111), (220), (311), (400), (511) and (440) facets (JSPDS01-1111, α = 8.393 Å), indicating inverse-spinel type structure of Fe_3_O_4_ NPs are synthesized. Moreover, no any other impure diffraction peaks were observed from the XRD patterns, suggesting that the as-prepared Fe_3_O_4_ NPs is pure Fe_3_O_4_.

**Figure 3:**
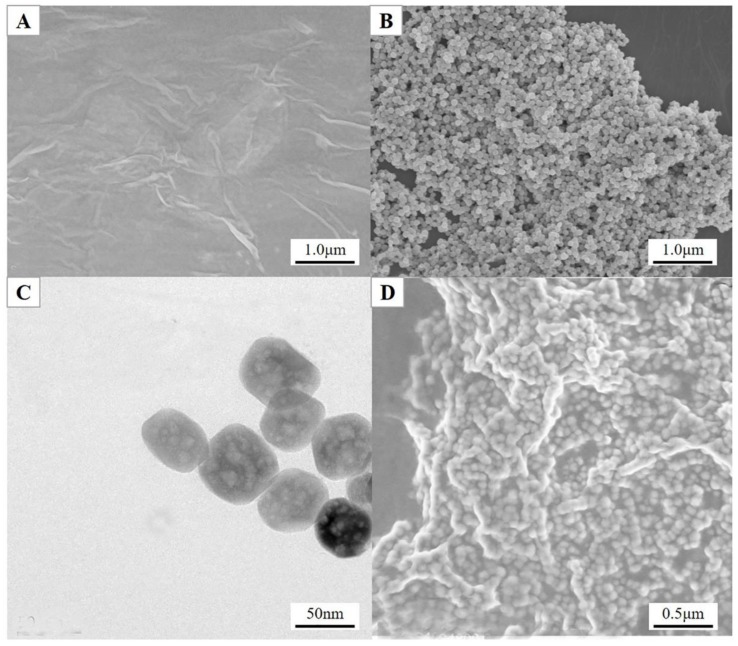
The Scanning Electron Microscopy (SEM) images of reduced graphene oxide (RGO) (**A**), NH_2_–Fe_3_O_4_ NPs (**B**) and NH_2_–Fe_3_O_4_/RGO nanocomposites (**D**); The Transmission Electron Microscopy (TEM) image of NH_2_–Fe_3_O_4_ NPs (**C**).

The surface morphologies of the RGO, NH_2_–Fe_3_O_4_ NPs and NH_2_–Fe_3_O_4_/RGO nanocomposites were characterized by SEM and TEM. A typical wrinkled and thin sheet-like character of RGO was seen from Figure 3A. A uniform size distribution of NH_2_–Fe_3_O_4_ NPs was found from the SEM (Figure 3B). More interestingly, the NH_2_–Fe_3_O_4_ NPs themselves have mesoporous structures on their surface (Figure 3C). The average particle size is estimated to be ca. 50 nm. Moreover, the surface of NH_2_–Fe_3_O_4_ NPs were successfully coated with the thin RGO nanosheets (Figure 3D).

### 3.3. Electrochemical Active Area

**Figure 4:**
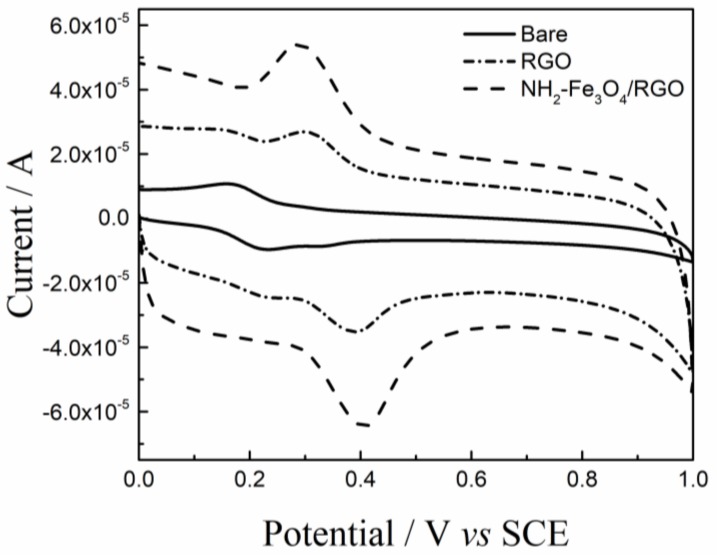
Cyclic voltammograms (CV) of the bare glassy carbon electrode (GCE), RGO/GCE and NH_2_–Fe_3_O_4_/RGO/GCE in 5 × 10^−4^ mol/L of [Fe(CN)_6_]^3−/4−^ solution. The CVs were recorded in 0.1 mol/L PBS (pH 3.5) at the scan rate of 100 mV/s.

The CVs recorded on the bare GCE, RGO/GCE and NH_2_–Fe_3_O_4_/RGO/GCE in 5 × 10^−4^ [Fe(CN)_6_]^3−/4−^ probe solution were shown in Figure 4. The reduction peak currents (*i_pc_*) on the bare GCE, RGO/GCE and NH_2_–Fe_3_O_4_/RGO/GCE are 8.284 × 10^−6^ A, 1.210 × 10^−5^ A and 2.924 × 10^−5^A, respectively. According to the Randles-Sevcik equation [43]:

(1) $$i_{pc}=2.69\times 10^{5}n^{3/2}D^{1/2}v^{1/2}AC$$

where *i_pc_* is reduction peak current of K_3_Fe(CN)_6_; *n* is the electron transferred number; *A* is electrochemical active area (cm^2^); *D* is the diffusion coefficient of K_3_Fe(CN)_6_ (*D* = 7.6 × 10^−6^cm^2^s^−1^ [44]); *C* is the concentration of K_3_[Fe(CN)_6_] (mol/cm^3^); *v* is the scan rate (V/s). The electrochemical active areas of the bare GCE, RGO/GCE and NH_2_–Fe_3_O_4_/RGO/GCE are 0.067 cm^2^, 0.103 cm^2^ and 0.249 cm^2^, respectively. The calculated area is well coincided with the geometric area (Φ 3.0 mm, 0.071 cm^2^) for bare GCE. The electrochemical active areas of NH_2_–Fe_3_O_4_/RGO/GCE is 3.7 times as compared to the bare GCE, indicating that the NH_2_–Fe_3_O_4_/RGO nanocomposites increase greatly the electrochemical active area.

### 3.4. The Electrochemical Behavior of Modified Electrodes

**Figure 5:**
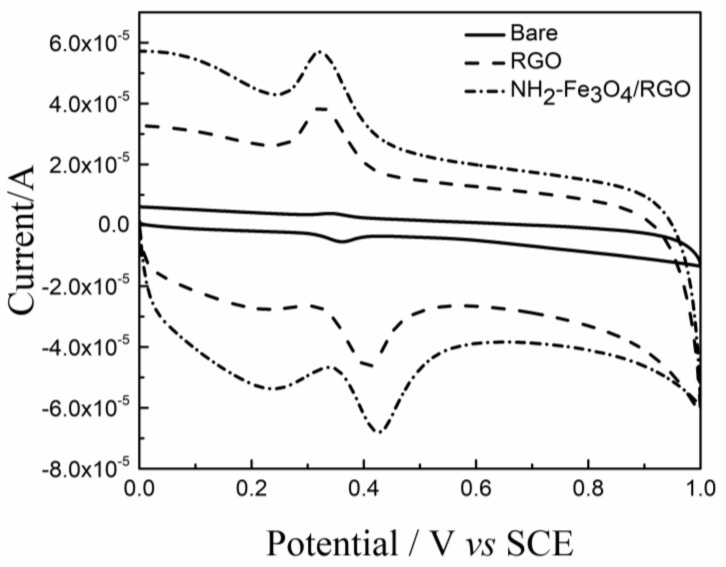
CVs of 1 × 10^−5^ mol/L DA on bare GCE, RGO/GCE and NH_2_–Fe_3_O_4_/RGO/GCE recorded in the presence of 0.1 mol/L PBS (pH 3.5) as supporting electrolyte. Scan rate: 100 mV/s.

The CV behaviors of DA (1.0 × 10^−5^ mol/L) on the surface of the bare and modified GCEs are shown in Figure 5. A pair of broad and weak redox peaks is observed on the bare GCE, indicating the electrochemical performance is poor. In this case, the oxidation peak current (*i_pa_*) of DA is 2.3 × 10^−6^ A. A pair of well-defined redox peaks appears on the RGO/GCE and the *i_pa_* (2.2 × 10^−5^ A) is approximately an order of magnitude higher than that on the bare GCE. The significant increase of *i_pa_* on the RGO/GCE is mainly due to good electrical conductivity and large electrochemical active area of RGO. Moreover, the π-π interactions between RGO and DA promote the charge transfer and DA adsorption process. When the NH_2_–Fe_3_O_4_/RGO/GCE acted as working electrode, the redox peaks are sharp and reversible and the *i_pa_* (2.9 × 10^−5^A) is nearly 13 times as compared to that on the bare GCE, due to the synergetic effect between mesoporous NH_2_–Fe_3_O_4_ and RGO sheets. These results confirmed that NH_2_–Fe_3_O_4_/RGO with much higher electrocatalytic activity toward the oxidation of DA.

### 3.5. Optimization of the Detection Conditions of DA

#### 3.5.1. The Influence of pH Value

**Figure 6:**
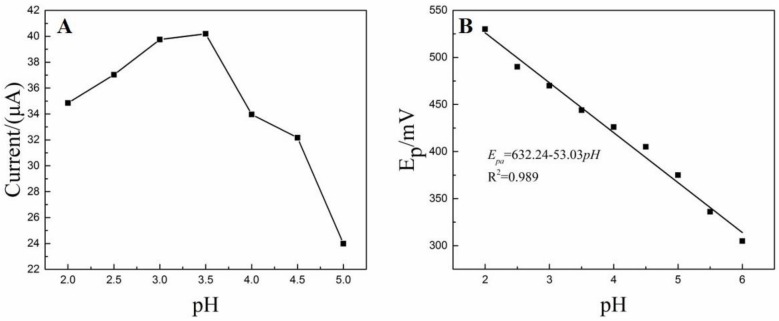
(**A**) The effect of pH on the oxidation peak currents of dopamine (DA); (**B**) Linear relationship between oxidation peak potentials (*E_p_*) and pH.

The electrochemical oxidation of DA is strong dependent upon the pH value, so it deserves further investigation the effect of pH on the oxidation peak current. Plot of oxidation peak currents (*i_pa_*) versus pH is presented in Figure 6A. It is obvious that the *i_pa_* increases with the increase of pH and highest *i_pa_* is obtained with pH of 3.5. Afterwards, the *i_pa_* gradually decreases when the pH increases further from 3.5 to 5.0. Thus, the pH of 3.5 was chosen for the subsequent experiments. Furthermore, peak potential (*E_p_*) shifted negatively with the increase of pH (Figure 6B).The linear equation relating *E_p_* to pH is *E_p_* (mV) = −53.03 pH + 632.24 (*R*^2^ = 0.989) and the slope is −53.03 mV/pH. According to Nernst equation, the slope (−53.03) suggests the oxidation of DA is an equal number of electrons and protons transferred electrochemical process [45].

#### 3.5.2. The Influence of Scan Rate

**Figure 7:**
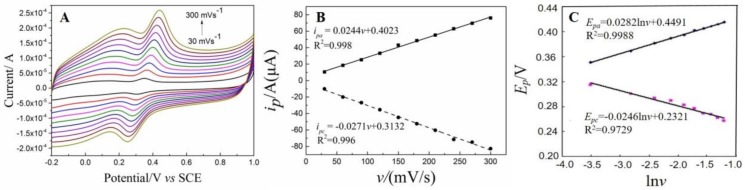
(**A**) CVs of 1 × 10^−5^ mol/L DA on the NH_2_–Fe_3_O_4_/RGO/GCE measured in 0.1mol/L phosphate buffer solution (PBS) at various scan rates (*v*); (**B**) Plot of redox peak currents (*i_p_*) versus scan rates (*v*); (**C**) Plots of redox peak potentials (*E_p_*) versus scan rates (*v*).

The scan rate is an important parameter that influences the sensing performance of DA. The CVs of 1 × 10^−5^ mol/L of DA on the NH_2_–Fe_3_O_4_/RGO/GCE recorded at various scan rates are presented in Figure 7A. Both *i_pa_* and *i_pc_* increased obviously with the increase of scan rates. Meanwhile, the background currents also increase with the increase of scan rates. In order to enhance signal to noise ratio (SNR) and reduce the background currents, 100 mV/s was selected for subsequent experiments. As shown in Figure 7B, the redox currents (*i_pa_* and *i_pc_*) is well linear to scan rates (*v*) and the linear equations are expressed as: *i_pa_* = 0.0244*v* + 0.4023 (*R*^2^ = 0.998) and *i_pc_* = −0.0271*v* + 0.3132 (*R*^2^ = 0.996), respectively. These results suggest that the electrochemical oxidation of DA on the NH_2_–Fe_3_O_4_/RGO/GCE is an adsorption-controlled process [13]. Thus, accumulation step is adopted to increase the response peak currents for subsequent experiments.

**Figure 8:**
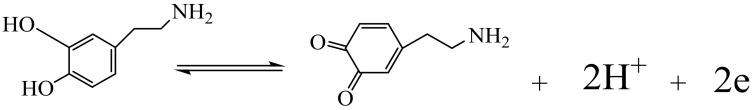
Scheme of electrochemical redox mechanism of DA.

In addition, *i_pa_* shifts positively with the increase of scan rates (*v*) while *i_pc_* shifts to negative direction, demonstrating that the oxidation of DA is quasi-reversible process. As shown in Figure 7C, the redox peak potentials (*E_pa_* and *E_pc_*) are linear to Napierian logarithm of scan rate (ln*v*). The linear equations are *E_pa_* = 0.0282 ln*v* + 0.449 (*R*^2^ =0.999) and *E_pc_* = −0.0246 ln*v* + 0.232 (*R*^2^ = 0.973), respectively. According to Lavrion equation [46]:

(2) $$E_{p}=E^{0}+\frac{RT}{\alpha nF}\ln\frac{RTk^{0}}{\alpha nF}+\frac{RT}{\alpha nF}\ln v$$

where *E^0^* is formal potential (V), *T* is Kelvin temperature (K), *α* is charge transfer coefficient, *n* number of electron transfer, *k^0^* is heterogeneous electron transfer rate, *F* is Faraday constant (96,480 C·mol^−1^), *R* is ideal gas constant (8.314 J·mol^−1^·K^−1^). As for a quasi-reversible process, *α* is generally assumed to be 0.5. Then *n* is calculated to be around 2. So, the oxidation of DA is two electrons and two protons transferred quasi-reversible process. The electrochemical redox mechanism of DA is summarized in Figure 8.

#### 3.5.3. Effect of Accumulation Potential and Time

**Figure 9:**
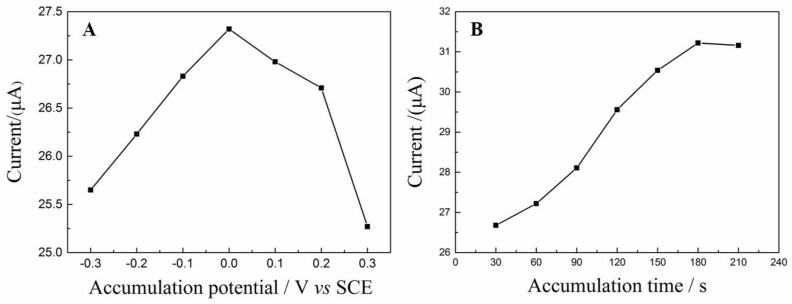
Effect of accumulation potential and time. (**A**) the oxidation peak currents (*i_pa_*) of DA after 210 s accumulation at various accumulation potential; (**B**) the oxidation peak currents (*i_pa_*) of DA after accumulation at 1.5 V for 30–210 s.

Before the detection of DA, accumulation was carried out to increase the concentration of DA on the surface of NH_2_–Fe_3_O_4_/RGO/GCE. The effect of accumulation conditions (accumulation potential and time) on the oxidation peak currents (*i_pa_*) was also investigated. The *i_pa_* of DA was measured after a 210 s accumulation step with various accumulation potentials. As shown in Figure 9A, the *i_pa_* of DA are highly dependent on the accumulation potential and the highest *i_pa_* appears when the accumulation potential is 0 V. Moreover, the effect of accumulation time on the *i_pa_* of DA was also investigated when the accumulation potentials were fixed to 0 V (Figure 9B). The *i_pa_* increases gradually with the increase of accumulation time within the first 180 s. Afterwards the *i_pa_* is almost unaltered with the further increase of accumulation time, suggesting that the adsorption achieved saturation rapidly. Thus, 0 V and 210 s is chosen for further experiments.

### 3.6. Interference Studies

**Figure 10:**
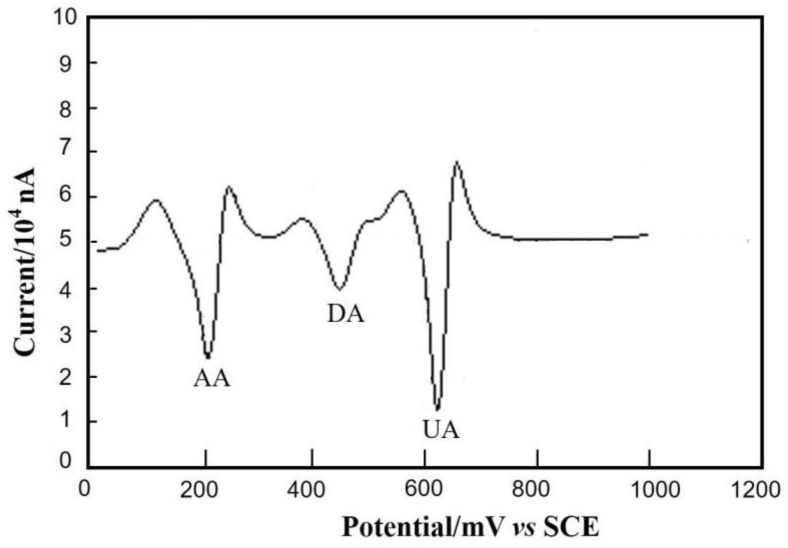
The Second-order derivative linear sweep voltammetry (SDLSV) of DA obtained on the NH_2_–Fe_3_O_4_/RGO/GCE in the mixture of ascorbic acid (AA) (1 × 10^−5^ mol/L), DA (2 × 10^−5^ mol/L) and uric acid (UA) (1 × 10^−5^ mol/L). Scan potential range: 0–1.0 V; scan rate: 100 mV/s; supporting electrolytes: 0.1 mol/L PBS.

High selectivity is critical to the detection of real samples since DA often coexists with AA and UA in blood samples. In this section, the electrochemical responses of DA (2 × 10^−5^ mol/L), AA (1 × 10^−5^ mol/L) and UA (1 × 10^−5^ mol/L) are investigated by SDLSV method. As shown in Figure 10, the response peaks of AA, DA and UA separate well each other and the peak potentials of AA, DA and UA are 216 mV, 444 mV and 620 mV, respectively. The peak potential differences (∆*E_p_*) of AA-DA and DA-UA are 228 mV and 176 mV, respectively. Moreover, the response peak currents of DA were not interfered even in the excess of AA and UA. This result indicates that the synergistic effect between NH_2_-Fe_3_O_4_ NPs and RGO improve the selectivity and anti-interference property significantly.

### 3.7. Calibration Curve, Linear Range and Detection Limit

**Figure 11:**
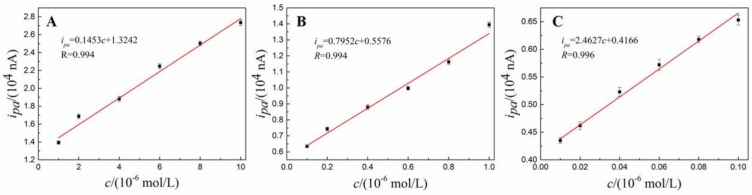
Calibration curves between response peak currents and the concentration of DA. The concentration ranges are 1 × 10^−8^ – 1 × 10^−7^ mol/L (**A**), 1 × 10^−7^ – 1 × 10^−6^ (**B**) and 1 × 10^−6^ – 1 × 10^−5^ mol/L (**C**).

The calibration curve, linear range and detection limit was investigated by SDLSV under optimal detection condition. Three linear regions are observed in the ranges of 1 × 10^−8^ – 1 × 10^−7^ mol/L(Figure 11A), 1 × 10^−7^ – 1 × 10^−6^ mol/L (Figure 11B) and 1×10^−6^ – 1×10^−5^ mol/L (Figure 11C). The linear equations are *i_pa_* = 0.1453*c* + 1.3242 (*R* = 0.994), *i_pa_* = 0.7952*c* + 0.5576 (*R* = 0.994) and *i_pa_* = 2.4627*c* + 0.4166 (*R* = 0.996), respectively. Where *i_pa_* (10^4^ nA) is oxidation peak currents, *c* (10^−6^ mol/L) is the concentration of DA. The detection limit (*S*/*N* = 3) is (4.0 ± 0.36) × 10^−9^ mol/L. The sensing performance in terms of linear range and detection limit as comparable even better than previous reports [24,35,47,48,49,50,51,52,53] as listed in Table 1. As we expected, our proposed sensor outperforms than the similar sensor (Fe_3_O_4_–NH_2_@GS/GCE) reported in literature [24]. This phenomenon can be well explained by reasons as follows. Firstly, the superior electrochemical performance of electrochemically reduced graphene oxide can be obtained under the optimal electrochemical reduction conditions. In contrast, the oxygen-containing groups that play curial role in electrochemical oxidation of dopamine have almost been removed due to drastic and uncontrollable chemical reduction process. Moreover, SDLSV have better sensitivity and selectivity compared with differential pulse voltammetry (DPV) reported reference [23], since SDLSV yields better signal-to-background.

### 3.8. Analysis of Real Samples

The practicability of NH_2_–Fe_3_O_4_/RGO/GCE was validated using SDLSV under the optimal detection conditions. As listed in Table 2, satisfactory results are obtained on the NH_2_–Fe_3_O_4_/RGO/GCE. The determination values of DA are well consistent with standard values with the RSD of −1.63~2.20% and the recovery rate is 97.1~103.9%. These results indicate that the NH_2_–Fe_3_O_4_/RGO/GCE have tremendous application prospects on the sensitive detection of DA in various real DA samples.

## 4. Conclusions

In summary, the NH_2_–Fe_3_O_4_/RGO/GCE are successfully prepared via drop-casting the NH_2_–Fe_3_O_4_/RGO dispersion on the bare GCE, followed by an electrochemical reduction of GO process. The optimal reduction potential is −1.5 V and reduction time is 120 s for electrochemical reduction of GO. The NH_2_–Fe_3_O_4_/RGO nanocomposites not only have the inherited advantages from the component materials but also improve properties due to synergetic effect between NH_2_–Fe_3_O_4_ NPs and RGO. As a result, the electrochemical active area of NH_2_–Fe_3_O_4_/RGO/GCE increased greatly as compared to those of bare GCE and RGO/GCE. A pair of well-shaped redox peaks is observed on the NH_2_–Fe_3_O_4_ NPs/RGO/GCE, suggesting the oxidation of DA is a quasi-reversible process. The electrochemical oxidation of DA on the NH_2_–Fe_3_O_4_/RGO/GCE is a two-electrons, two-protons transferred and adsorption-controlled process. The wide linear range (1 × 10^−8^ – 1 × 10^−7^ mol/L, 1 × 10^−7^ – 1 × 10^−6^ mol/L and 1 × 10^−6^ – 1 × 10^−5^ mol/L) and low detection limit (4.0 × 10^−9^ mol/L) are also obtained. Moreover, the response peak currents of DA were not interfered by the coexistence species such as AA and UA. Finally, the proposed NH_2_–Fe_3_O_4_/RGO/GCE are successfully applied to detect DA in real samples with satisfactory results.

## Figures and Tables

**Table 1:** A comparison of the sensing performance between NH_2_–Fe_3_O_4_/RGO/GCE and other modified electrodes reported in literatures.

Modified Electrodes	Linear Range (M)	Detection Limit (M)	Ref.
NH_2_–Fe_3_O_4_/RGO/GCE	1 × 10^−8^~1 × 10^−7^; 1 × 10^−7^~1 × 10^−6^; 1 × 10^−6^~1 × 10^−5^	4.0 × 10^−9^	This work
Fe_3_O_4_–NH_2_@GS/GCE	2 × 10^−7^~3.8 × 10^−5^	1.26 × 10^−7^	[24]
Cu_2_O/RGO/GCE	1 × 10^−8^~1 × 10^−6^; 1 × 10^−6^~8 × 10^−5^	6.0 × 10^−9^	[35]
Pd/RGO/GCE	1 × 10^−6^~1.5 × 10^−4^	2.3 × 10^−7^	[47]
Fe_3_O_4_@Au/Gr/GCE	5 × 10^−7^~5 × 10^−5^	6.5 × 10^−7^	[48]
Fe_3_O_4_/RGO/CPE	2 × 10^−8^~5.8 × 10^−6^	6.5 × 10^−9^	[49]
MWCNT/RGO/GCE	2 × 10^−7^~4 × 10^−4^	2.2 × 10^−8^	[50]
Mn_3_O_4_–RGO/GCE	1 × 10^−6^~1.45 × 10^−3^	2.5 × 10^−7^	[51]
MnO_2_–RGO/GCE	6 × 10^−8^~1× 10^−3^; 1 × 10^−6^ ~8 × 10^−5^	1.0 × 10^−9^	[52]
MnO_2_ NR/RGO/GCE	5 × 10^−8^ ~ 4 × 10^−4^	1.0 × 10^−8^	[53]

**Table 2:** Determination results of the dopamine hydrochloride injections (*n* = 4).

No.	Standard Value (μM)	Determination Value (μM)	Added (μM)	Total Found (μM)	Recovery (%)	RSD (%)
1	13.14	12.85	10.00	23.24	103.9	2.20
2	27.63	27.18	30.00	56.85	98.9	−1.63
3	48.62	49.21	50.00	97.74	97.1	1.21

## References

[1] Liu A., Honma I., Zhou H. (2007). Simultaneous voltammetric detection of dopamine and uric acid at their physiological level in the presence of ascorbic acid using poly(acrylic acid)-multiwalled carbon-nanotube composite-covered glassy-carbon electrode. Biosens. Bioelectron..

[2] Liu A., Honma I., Zhou H. (2005). Amperometric biosensor based on tyrosinase-conjugated polysacchride hybrid film: Selective determination of nanomolar neurotransmitters metabolite of 3, 4-dihydroxyphenylacetic acid (DOPAC) in biological fluid. Biosens. Bioelectron..

[3] He Q., Liu J., Liang J., Liu X., Li W., Liu Z., Ding Z., Tuo D. (2018). Towards improvements for penetrating the blood–brain barrier—recent progress from a material and pharmaceutical perspective. Cells.

[4] Zhou Y., Yan H., Xie Q., Huang S., Liu J., Li Z., Ma M., Yao S. (2013). Simultaneous analysis of dopamine and homovanillic acid by high-performance liquid chromatography with wall-jet/thin-layer electrochemical detection. Analyst.

[5] Lin L., Qiu P., Yang L., Cao X., Jin L. (2006). Determination of dopamine in rat striatum by microdialysis and high-performance liquid chromatography with electrochemical detection on a functionalized multi-wall carbon nanotube electrode. Anal. Bioanal. Chem..

[6] Hows M.E., Lacroix L., Heidbreder C., Organ A.J., Shah A.J. (2004). High-performance liquid chromatography/tandem mass spectrometric assay for the simultaneous measurement of dopamine, norepinephrine, 5-hydroxytryptamine and cocaine in biological samples. J. Neurosci. Methods.

[7] Govindaraju S., Ankireddy S.R., Viswanath B., Kim J., Yun K. (2017). Fluorescent gold nanoclusters for selective detection of dopamine in cerebrospinal fluid. Sci. Rep..

[8] Chibac A.L., Melinte V., Buruiana T., Buruiana T., Buruiana E.C. (2017). Fluorescent polymeric sensors containing boronic acid derivatives for sugars and dopamine detection: Sensing characteristics enhancement by Au NPs. Sens. Actuators B.

[9] Wu B., Miao C., Yu L., Wang Z., Huang C., Jia N. (2014). Sensitive electrochemiluminescence sensor based on ordered mesoporous carbon composite film for dopamine. Sens. Actuators B.

[10] Zhang L., Cheng Y., Lei J., Liu Y., Hao Q., Ju H. (2013). Stepwise chemical reaction strategy for highly sensitive electrochemiluminescent detection of dopamine. Anal. Chem..

[11] Ensafi A.A., Taei M., Khayamian T., Arabzadeh A. (2010). Highly selective determination of ascorbic acid, dopamine, and uric acid by differential pulse voltammetry using poly(sulfonazo III) modified glassy carbon electrode. Sens. Actuators B.

[12] Revin S.B., John S.A. (2012). Highly sensitive determination of uric acid in the presence of major interferents using a conducting polymer film modified electrode. Bioelectrochemistry.

[13] Ciszewski A., Milczarek G. (1999). Polyeugenol-Modified Platinum Electrode for Selective Detection of Dopamine in the Presence of Ascorbic Acid. Anal. Chem..

[14] Feng X., Mao C., Yang G., Hou W., Zhu J. (2006). Polyaniline/Au Composite Hollow Spheres:  Synthesis, Characterization, and Application to the Detection of Dopamine. Langmuir.

[15] Shams E., Babaei A., Taheri A.R., Kooshki M. (2009). Voltammetric determination of dopamine at a zirconium phosphated silica gel modified carbon paste electrode. Bioelectrochemistry.

[16] Zare H.R., Sobhani Z., Mazloum-Ardakani M. (2006). Electrochemical behavior of electrodeposited rutin film on a multi-wall carbon nanotubes modified glassy carbon electrode. Improvement of the electrochemical reversibility and its application as a hydrazine sensor. J. Solid State Electrochem..

[17] Liu Y., Huang J., Hou H., You T. (2008). Simultaneous determination of dopamine, ascorbic acid and uric acid with electrospun carbon nanofibers modified electrode. Electrochem. Commun..

[18] Wen J., Zhou L., Jin L., Cao X., Ye B. (2009). Overoxidized polypyrrole/multi-walled carbon nanotubes composite modified electrode for in vivo liquid chromatography–electrochemical detection of dopamine. J. Chromatogr. B.

[19] He Q., Liu J., Huang C., Wu W. (2014). Synthesis and Characterization of a Silver Incorporated Magnetic Nanocomposite with Enhanced Antibacterial Activity. Sci. Adv. Mater..

[20] He Q., Liu J., Huang C., Wu Z. (2014). A nanoscale system for remarkably enhanced drug delivery based on hollow magnetic particles encapsulated within temperature-responsive poly(methylmethacrylate). Sci. Adv. Mater..

[21] Liu J., Huang C., He Q. (2015). Pharmaceutical application of magnetic iron oxide nanoparticles. Sci. Adv. Mater..

[22] He Q., Liu J., Liang J., Liu X., Tuo D., Li W. (2018). Chemically Surface Tunable Solubility Parameter for Controllable Drug Delivery—An Example and Perspective from Hollow PAA-Coated Magnetite Nanoparticles with R6G Model Drug. Materials.

[23] He Q., Liu J., Liang J., Liu X., Ding Z., Tuo D., Li W. (2018). Sodium Acetate Orientated Hollow/ Mesoporous Magnetite Nanoparticles: Facile Synthesis, Characterization and Formation Mechanism. Appl. Sci..

[24] Wu D., Li Y., Zhang Y., Wang P., Wei Q., Du B. (2014). Sensitive electrochemical sensor for simultaneous determination of dopamine, ascorbic acid, and uric acid enhanced by amino-group functionalized mesoporous Fe_3_O_4_@graphene sheets. Electrochim. Acta.

[25] Chen R., Song G., Wei Y. (2010). Synthesis of variable-sized Fe_3_O_4_ nanocrystals by visible light irradiation at room temperature. J. Phys. Chem. C.

[26] Ye Y., Kong T., Yu X., Wu Y., Zhang K., Wang X. (2012). Enhanced nonenzymatic hydrogen peroxide sensing with reduced graphene oxide/ferroferric oxide nanocomposites. Talanta.

[27] Compton O.C., Nguyen S.T. (2010). Graphene oxide, Highly reduced graphene oxide, and graphene: Versatile building blocks for carbon-based materials. Small.

[28] Rani G.J., Babu K.J., Gnana kumar G., Rajan M.A.J. (2016). Watsonia meriana flower like Fe_3_O_4_/reduced graphene oxide nanocomposite for the highly sensitive and selective electrochemical sensing of dopamine. J. Alloys Compd..

[29] Wang Y., Zhang H., Yao D., Pu J., Zhang Y., Gao X., Sun Y. (2013). Direct electrochemistry of hemoglobin on graphene/Fe_3_O_4_ nanocomposite-modified glass carbon electrode and its sensitive detection for hydrogen peroxide. J. Solid State Electrochem..

[30] Rocha-Santos T.A.P. (2014). Sensors and biosensors based on magnetic nanoparticles. Trends Anal. Chem..

[31] Wang Y., Liu Q., Qi Q., Ding J., Gao X., Zhang Y., Sun Y. (2013). Electrocatalytic oxidation and detection of N-acetylcysteine based on magnetite/reduced graphene oxide composite-modified glassy carbon electrode. Electrochim. Acta.

[32] Xin X., Wei Q., Yang J., Yan L., Feng R., Chen G., Du B., Li H. (2012). Highly efficient removal of heavy metal ions by amine-functionalized mesoporous Fe_3_O_4_ nanoparticles. Chem. Eng. J..

[33] Chang Y., Ren C., Qu J., Qu J., Chen X. (2012). Preparation and characterization of Fe_3_O_4_ /graphene nanocomposite and investigation of its adsorption performance for aniline and p-chloroaniline. Appl. Surf. Sci..

[34] Sun X., Yang L., Li Q., Zhao J., Li X., Wang X., Liu H. (2014). Amino-functionalized magnetic cellulose nanocomposite as adsorbent for removal of Cr(VI): Synthesis and adsorption studies. Chem. Eng. J..

[35] He Q., Liu J., Liu X., Li G., Deng P., Liang J. (2018). Preparation of Cu_2_O-reduced graphene nanocomposite modified electrodes towards ultrasensitive dopamine detection. Sensors.

[36] Dreyer D.R., Park S., Bielawski C.W., Ruoff R.S. (2010). The chemistry of graphene oxide. Chem. Soc. Rev..

[37] Guo H.L., Wang X.F., Qian Q.Y., Wang F.B., Xia X.H. (2009). A green approach to the synthesis of graphene nanosheets. ACS Nano.

[38] Xiong H., Jin B. (2011). The electrochemical behavior of AA and DA on graphene oxide modified electrodes containing various content of oxygen functional groups. J. Electroanal. Chem..

[39] Deng P., Fei J., Feng Y. (2011). Sensitive voltammetric determination of tryptophan using an acetylene black paste electrode modified with a Schiff’s base derivative of chitosan. Analyst.

[40] Hummers J.W.S., Offeman R.E. (1958). Preparation of graphitic oxide. J. Am. Chem. Soc..

[41] Pei S., Cheng H.M. (2012). The reduction of graphene oxide. Carbon.

[42] Sivasubramanian R., Biji P. (2016). Preparation of copper (I) oxide nanohexagon decorated reduced graphene oxide nanocomposite and its application in electrochemical sensing of dopamine. Mater. Sci. Eng. B.

[43] Bard A.J., Faulkner L.R. (2000). Electrochem Methods: Fundamentals and Application.

[44] Gooding J., Praig V., Hall E. (1998). Platinum-catalyzed enzyme electrodes immobilized on gold using self-assembled layers. Anal. Chem..

[45] Deng P., Xu Z., Kuang Y. (2013). Electrochemically reduced graphene oxide modified acetylene black paste electrode for the sensitive determination of bisphenol A. J. Electroanal. Chem..

[46] Laviron E. (1979). General expression of the linear potential sweep voltammogram in the case of diffusionless electrochemical systems. J. Electroanal. Chem. Interfacial Electrochem..

[47] Palanisamy S., Thirumalraj B., Chen S.M. (2015). Palladium nanoparticles decorated on activated fullerene modified screen printed carbon electrode for enhanced electrochemical sensing of dopamine. J. Colloid Interf. Sci..

[48] Liu M., Chen Q., Lai C., Zhang Y., Deng J., Li H., Yao S. (2013). A double signal amplification platform for ultrasensitive and simultaneous detection of ascorbic acid, dopamine, uric acid and acetaminophen based on a nanocomposite of ferrocene thiolate stabilized Fe_3_O_4_@Au nanoparticles with graphene sheet. Biosens. Bioelectron..

[49] Bagheri H., Afkhami A., Hashemi P., Ghanei M. (2015). Simultaneous and sensitive determination of melatonin and dopamine with Fe_3_O_4_ nanoparticle-decorated reduced graphene oxide modified electrode. RSC Adv..

[50] Cheemalapati S., Palanisamy S., Mani V., Chen S.M. (2013). Simultaneous electrochemical determination of dopamine and paracetamol on multiwalled carbon nanotubes/graphene oxide nanocomposite-modified glassy carbon electrode. Talanta.

[51] Yao Z., Yang X., Niu Y., Wu F., Hu Y., Yang Y. (2017). Voltammetric dopamine sensor based on a gold electrode modified with reduced graphene oxide and Mn_3_O_4_ on gold nanoparticles. Microchim. Acta.

[52] He Q., Liang J., Li G., Deng P., Liu J., Liu X. (2018). Electrochemical detection of dopamine based on MnO_2_ nanowires/reduced graphene oxide composites modified glassy carbon electrode. Chinese J Anal. Chem..

[53] Wang Z., Tang J., Zhang F. (2013). Elimination of ascorbic acid and sensitive detection of uric acid at the MnO_2_ nanorods/graphene-based modified electrode. Int. J. Electrochem. Sci..

